# Cryo-attenuated properties of *Tilia miqueliana* pericarps and seeds

**DOI:** 10.3389/fpls.2023.1228069

**Published:** 2023-08-15

**Authors:** Yu Wu, Xiao Rui Sun, Chen Yin Peng, Yong Bao Shen, Anne M. Visscher, Hugh W. Pritchard, Ming Zhu Wang, Zhi Yun Deng

**Affiliations:** ^1^ College of Forestry, Nanjing Forestry University, Nanjing, Jiangsu, China; ^2^ Co-innovation Center for Sustainable Forestry in Southern China, Southern Tree Inspection Center National Forestry Administration, Nanjing, Jiangsu, China; ^3^ Royal Botanic Gardens, Kew, Ardingly, United Kingdom; ^4^ Kunming Institute of Botany, Chinese Academy of Sciences, Kunming, Yunnan, China

**Keywords:** linden, hard pericarp and seeds, LN treatment, cryo-attenuated properties, postharvest and deep processing

## Abstract

**Introduction:**

Cryo treatment of dry seeds is known to attenuate the structure of fruit and seed coats, but little is known about the microstructural impacts of such treatment. The seeds of *Tilia miqueliana* are dispersed within a hard pericarp, the manual removal (hulling) of which is time-consuming and inefficient. Rapid hulling technology is urgently needed for sustainable production and convenience of edible nuts.

**Methods:**

We explored the mechanistic basis of liquid nitrogen (N)-treatment weakening of the pericarp of *T. miqueliana* fruits using a range of microscopical, biophysical and chemical approaches.

**Results:**

Liquid N treatment (40 s) resulted in lower pericarp contents of cellulose and hemicellulose, and increased amounts of lignin. Profound changes in cell structure and mechanical properties included the emergence of large holes and gaps between the mesocarp and endocarp cells. Also, the toughness of the pericarp decreased, whilst the hardness and brittleness increased, thereby changing the fracture type from ductile to brittle. Liquid N treatment of dry fruits followed by tapping with a hammer, reduced the number of damaged seeds three-fold and pericarp peeling time four-fold compared with manual hulling, whilst seed viability was not negatively affected.

**Discussion:**

Comparable findings for the efficient and economical removal of hard covering structures from dispersal units of five more species from three other families following liquid N treatment indicates the potential application of our findings to large-scale production of seeds and seedlings for breeding, forestry and conservation/restoration purposes. Furthermore, it introduces a novel concept for postharvest treatment and pre-treatment of deep processing in nuts.

## 1 Introduction

Even when water enters the interior of some seeds with hard shells, germination may still be delayed as the hard shell remains an obstacle to emergence. *Tilia miqueliana* is a representative species with this type of seed surrounded by a tough pericarp. In terms of dormancy type, this species is considered to have a combination of mechanical + physiological dormancy. The pericarp and seed coat impose a mechanical constraint and the seed’s physiological dormancy is caused by the endosperm (Shi, 2006).

*T. miqueliana* is a species in the *Malvaceae* family (formerly *Tiliaceae*). The unique large leaf bracts on its inflorescence are highly ornamental. It is an excellent wood and ornamental tree species, and its flowers are favored by honeybees. In Europe, scented tea made from linden flowers is used to prevent influenza and is also the most commonly consumed beverage (Pavlović et al., 2020). The linden tree (any of the species *Tilia x europaea*, *Tilia cordata*, *Tilia platyphyllos*, *Tilia americana* etc.) has fruit that contain nut-like seeds. These have a range of essential oils and many of these have properties that make them taste like chocolate. It wasn’t until the 18th century that people began to experiment with linden fruit to try and make something more palatable from it. As a result, the French chemist, Missa discovered that by grinding the immature fruit of linden trees with dried linden flowers he could obtain a product that had an aroma similar to chocolate. It is also a symbol of Buddhist culture. The fullness rate of the populations is around 50%, while the isolated does not exceed 5% (Shi, 2006; Gao, 2008). Moreover, the seeds are deeply dormant and need 2–3 years to germinate under natural conditions (Shi et al., 2012). These factors seriously affect the seed yield and seedling propagation of this species. At present, there are about 2000 wild *T. miqueliana* trees in China (Tang and Tang, 2007; Yan and Huang, 2021). Thus, it is an endangered and rare species under national protection (Shi et al., 2012).

Various methods (sulfuric acid + gibberellin + stratification) have been used to treat *T. miqueliana* fruit, but none have been shown to be particularly efficient at improving germination performance. This problem limits the use of the fruit as the germination unit for large-scale seedling production for species recovery or habitat restoration (Shi, 2006). Most of the seeds obtained by shelling can germinate quickly, particularly when combined with phytohormone treatment to alleviate physiological dormancy. For example, seeds treatment with 98% H_2_SO_4_ for 15 min, followed by washing vigorously with 500 mT magnetically treated water (MTW) for 24 h, then immersion in 500 mg·L^−1^ GA_3_ for 12 h, and finally stratified at 4 °C. It was found that 75% seeds germinated within 75 days (Yao et al., 2015). Therefore, seeds with the pericarps removed are currently used for the production of *T. miqueliana* seedlings in China.

The use of manual shelling of the pericarp to obtain the seed is time-consuming, inefficient, and it cannot meet the needs of large-scale breeding and production of *T. miqueliana* (Shi, 2006). Within the pericarp, the mechanical barrier is caused by a highly lignified and hardened mesocarp. Physical and chemical treatments can reduce the mechanical strength of these seed covering structures. Physical treatments include mechanical damage, low and high temperature treatments, dry and wet treatments, radiation, and high-pressure treatment. Chemical treatments include acid etching, lye immersion, and organic solvent treatment (Mousavi et al., 2011). All combinations of treatments need to impose physical changes without compromising the physiological viability of the seed contained therein. We developed a method of using liquid N treatment to remove pericarp, the results showed that treatment with liquid N makes the pericarp brittle and easy to crack, and that the germination rate of the seeds is negatively affected by this treatment. Comparative studies on another five commercial forestry species indicate that the liquid N-based method for hulling has widescale potential for use in breeding, forestry and conservation/restoration programmes.

## 2 Materials and methods

### 2.1 Plant materials

*T. miqueliana* fruits were harvested in November 2020 at the Huang Zangyu National Forest Park (117°03′–117°06′E, 34°–34°06′N), Anhui Province, China. The fruits were placed in net bags and stored at room temperature.

Fruits with full seeds were selected for testing based on X-ray image analysis. Fruits were placed on No. 3 enlarging paper and radiographed using an HY-35 X-ray machine (Xiang Xi Instrument Factory, Hunan, China) at a voltage of 80 kV, 5 mA electric current, 120 s exposure time and a focus-film-distance of 25 cm.

### 2.2 Removal of pericarps manually and using liquid N

The fruits were treated with liquid N (Group 1) or dehulled manually (Group 2) as follows:

Group 1: fruits were placed in a stainless-steel box, and liquid N was poured into the box (volume ratio of fruits: liquid N = 1:1.5). After 20, 40, 60, and 80 s in contact with liquid N (-196°C), the fruits were placed on the laboratory bench and the pericarp was tapped with a 2.6-cm diameter hammer.

Group 2: The fruits at room temperature were squeezed in a vice to break the pericarp.

For Group 1 and 2 treatments, four replicates of 100 fruits were used. The processing time for 100 fruits was recorded and used to estimate the average processing time per fruit. Finally, undamaged seeds were removed with tweezers while damaged seeds were discarded and the percentage of damaged seeds during hulling determined.

### 2.3 Seed germination test

Seeds collected in 2017, 2018, and 2019 were treated with liquid N for 40 s and tapped with a hammer to remove the pericarp. After seed removal, undamaged seeds were placed in a 50-mL beaker, and distilled water was added (volume ratio of seeds: distilled water = 1:2). The full seeds sinking to the bottom were removed and dried in air. All full seeds were placed into a 500-mL beaker. Acid (98% H_2_SO_4_) was added to the beaker (seed volume: acid ratio = 1:1.5) and then the mixture was stirred continuously for 15 min with a glass rod. Gauze was tied around the mouth of the beaker, and then all of the acid was poured off. The beaker containing the seeds was rinsed with tap water for 20 min and the seeds placed into a yarn bag and soaked in gibberellin (GA_3_) solution (500 mgL^−1^) in a 500-mL beaker (seed: GA volume ratio = 1:1.2). The beaker was wrapped in a black plastic bag and placed in the dark for 12 h, and then the GA_3_ solution was poured off. Seeds were mixed with wet sand (45% humidity) at a 1:3 (w/w) ratio and stratified at 4°C for 75 days in darkness. When the radicle length reached 2 mm, the seeds were considered to have germinated. Final germination is presented as mean ± SD, based on four replicates of 100 full seeds. Manually hulled seeds served as the control.

### 2.4 Preparation of paraffin sections

Paraffin sections were prepared and observed to characterize pericarp cell morphology. Eight samples were collected from the control (manually hulled) and liquid N (40 s) groups. An 1-mm^3^ piece was cut from the pericarp of each fruit, and then fixed for 24 h in a 70% formalin-acetic acid-alcohol fixative solution (Servicebio, Wuhan, China). Samples were then dehydrated, using an ethanol series, and embedded in a wax block. The wax blocks were cut into 10-μm sections using an HistoCore autocut sliding microtome (Leica, Guangzhou, China) equipped with a tungsten steel slicer. The sections were stained with safranine and fast green. All sections were scanned using a section scanner (Panoramic desk/midi/250/1000, 3DHISTECH Company, Budapest, Hungary). The images were viewed at 1× to 400× magnification using CaseViewer 2.4 software (https://www.3dhistech.com/solutions/caseviewer/). Target areas of the tissue was selected for 200× imaging. The areas of lignin and cellulose were measured using Image-Pro Plus 6.0 analysis software (two-and three-dimensional image processing and analysis) using mm as the standard unit length. The area percentages of lignin and cellulose were calculated based on 100 fields of view (each being 0.48864 mm^2^).

### 2.5 Scanning electron microscopy analyses of pericarps

Eight fruit coat samples were collected from the control and liquid N (40 s) groups. A 2-mm^2^ piece of the pericarp was mounted with double-sided tape on the sample stage of a scanning electron microscopy (SEM) (Thermo Fisher Scientific, Waltham, MA, USA). Samples were then gold-coated using a gold sputter coater (HITACHI E-1010, Tokyo, Japan) and observed by SEM in the high vacuum mode. Images were captured at 15 kV.

### 2.6 Atomic force microscopy

Atomic force microscopy (AFM) (Dimension Edge, Bruker, Germany) was used to observe the 3D structure of the ventral suture of the pericarp. Eight fruit samples were collected from the control and liquid N (40 s) groups. Tissue from the carpel junction of the pericarp was cut into 1-mm^3^ pieces and fixed immediately in 2.5% v/v glutaraldehyde aqueous fixative solution (Servicebio) for 2 h, then transferred to 1% w/v OsO_4_ for 5 h, dehydrated in an ethanol series, and embedded in resin blocks. The resin blocks were cut using an ultra-thin microtome (Leica UC7, Wetzlar, Germany) equipped with a diamond slicing knife (Ultra 45°, Daitome, Bienne, Switzerland). The resin slices were fixed onto slides with double-sided tape.

Atomic level profile topography images by controlled probe piercing of the sample surface were acquired at a scan rate of 1.0 Hz and scan range of 5 µm.

### 2.7 Determination of lignin, cellulose, and hemicellulose contents of pericarps

Thirty pericarps from the control and liquid N (40 s) groups were analysed. The cellulose content was determined using the anthrone colorimetric method (Dubois et al., 1956); the lignin content by the acetyl bromide spectrophotometric method (Fukushima and Hatfield, 2001); and the hemicellulose content the by 3, 5-dinitrosalicylic acid (DNS) assay (Deshavath et al., 2020).

### 2.8 Determination of total sugar and sugar components in pericarps

To determine the total sugar contents, the dry pericarp (200 mg for each of 6 replicates) was repeatedly extracted (four times) with 20 mL 80% ethanol at room temperature and centrifuged at 10,000 × g for 20 min. The supernatant was collected and evaporated at 50°C under vacuum. The residue was dissolved to 20 mL with distilled water with 0.5 g poly (vinypolypyrrolidone), and then the mixture was centrifuged at 10,000 x g for 10 min. The supernatant was made up to a known volume and was assayed for total sugar by the dinitrosalicylic acid method (Gong et al., 2014). Total sugar content (%) = (C × V × N)/(10^6^ ×V_S_ × W) ×100, where C = sugar content converted from standard curve (μg), V = total volume of constant volume solution (mL), N = dilution multiple, Vs = volume of constant solution absorbed during determination (mL), and W = weight of sample (g).

Gas chromatography-mass spectrometry (GC-MS; 7890A-5975C, Agilent, American) was used to determine the content of six sugar components (arabinose, fructose, glucose, sorbitol, sucrose, and trehalose) in pericarps (Bianchi et al., 1993). The dry pericarp was extracted with 1 mL of 80% methanol and concentrated by drying. An aliquot of 30 µL of pyridinium methoxylate hydrochloride solution and 70 µL N, O-bis (trimethylsilyl) trifluoroacetamide was added to the dried sample before centrifugation. The supernatant was analysed by GC-MS using a HP-5 column (30m × 0.25mm × 0.25 µm). The transfer line temperature was 280 °C and the ion trap temperature 220°C. Other conditions for GC and MS were as follows: injector temperature: 220°C; detector temperature: 28°C; scanning range (m/z): 50-1000 amu at a rate of 1.5 scan/s; electron ionization energy: 70eV. The substance types under different peaks were searched in the NIST database through the data processing system of the X-caliber workstation to determine each sugar component. The relative content of each component (%) was calculated by area normalization, thus: Content of each sugar component (%) = (peak area of each sugar component)/sum of peak areas of sugar components × 100.

### 2.9 Hardness and elastic modulus of the carpel junction of the pericarp

Nanoindentation is a relatively new technique that has been used to measure the nanomechanical properties of surface layers of bulk materials and thin films (Tarefder et al., 2010). Eight seeds from the control and liquid N (40 s) groups were analysed. Samples collected from the carpel junction of the pericarp were cut into 1-mm^3^ pieces and fixed immediately in 2.5% v/v glutaraldehyde aqueous fixative solution (Servicebio) for 2 h, then transferred to 1% OsO_4_ w/v for 5 h, dehydrated in an ethanol series, and embedded in resin blocks. The resin blocks were sliced using an ultra-thin microtome equipped with a diamond slicing knife as described above. The surface of the pericarp was polished. A Nanoindenter (iMicro, Nanomechanics, Oak Ridge, TN, USA) was used to test the hardness and elastic modulus of the pericarp. The resin blocks were fixed on a round slide with glue. A Berkovich indenter was used in the ‘load control’ mode, with the loading rate/load constant kept constant during the loading process. The conditions were: a load resolution of 50 nN, displacement resolution of 0.01 nm, 0.4 mN maximum applied load, 100 μN/s loading and unloading rate, 150 nm pressure depth, 5 s duration at maximum load, and a Poisson’s ratio (nu) of 0.28. The optical microscope of the instrument was used to randomly choose the indentation area of 10 spots around the mesocarp. Each data item was the average of three test results. At the same time, the morphology of the indentation was observed. When calculating the elastic modulus and hardness by the Oliver & Pharr method (Li et al., 2020), the hardness (H) and equivalent modulus (Er) can be defined by the following formulae: H = (P_max_)/Ac and Er = (S × √π)/(2β× √Ac), where P_max_ is the maximum loading load, Ac is the surface area of the indentation, β is a parameter related to the shape of the indenter (for the Berkovich indenter, the value of β is 1.034), and S is the contact stiffness.

### 2.10 Crystallinity of the pericarp

An X-ray diffractometer (Ultima IV, Rigaku, Japan) was used to test the crystallinity of the pericarp. Four replicates of 30 seeds from the control and liquid N (40 s) groups were used in these analyses. The pericarps were incubated at 60°C for 4 h, and then crushed into a powder using a crusher (DE-300 g, Hongjingtian, China). The powder was put through a 320-mesh sieve, spread out in the sample preparation frame, and then pressed flat with a knife. The test parameters were as follows: Horizontal Goniometer, generator power = 3 KW, voltage = 40 kV, current = 30 mA. Crystallinity was calculated from X-ray diffraction (XRD) spectra using Jade 6.5 software. Crystallinity values were determined using the formula: Xcw = (Ic)/(Ic + Iw) × 100%, where Xcw is X crystallinity %, Ic is the intensity of the crystalline diffraction peak, and Iw is the intensity of the amorphous dispersion peak. The grain size of the crystal plane was calculated by Scherrer’s formula: D = (K×λ)/(COSθ × β), where K = Scherrer constant, D = average thickness of the crystal grain perpendicular to the crystal plane, B = half-height width of the diffraction peak of the sample, θ = diffraction angle, and γ = X-ray wavelength (0.154056 Å).

### 2.11 Hardness of the pericarp

The hardness of the pericarp was measured using a Vickers hardness machine (Falcon 507, Innovatest, Maastricht, The Netherlands). Eight seeds from the control and liquid N (40 s) groups were used in these analyses. After the outer surface of the pericarp was polished, the fruit was fixed with a clamp. A rhombus-shaped indentation was pressed into the pericarp’s outer surface using a diamond square pyramid with a vertex angle of 136°. A loading force of 10 gf was used and maintained for 10 s. The pressure was calculated based on the pressure per unit surface area of the indentation. The length of the two diagonal lines of the indentation was measured using the microscope on the instrument, and the software automatically displayed the hardness value.

### 2.12 Toughness of the pericarp

Pericarp toughness was evaluated at room temperature using a universal testing machine (WDW-10, Bairuo, China). Eight seeds from the control and liquid N (40 s) groups were analysed. The seed was fixed on the fulcrum. Force was applied to the surface of the pericarp until it broke, and the fracture toughness recorded. Measurements were conducted in ‘displacement control’ mode, and the loading rate was 3 mm/min. The crack opening displacement (COD) was measured using a YYJ-10/6-N clip extensometer. The morphology of the fracture surface was observed under a fluorescent stereo microscope (M205FA, Leica).

### 2.13 Brittleness of the pericarp

Brittleness was evaluated using a low temperature brittleness testing machine (JMDW-2C, Shanghai, China) in eight fruits from the control and liquid N (40 s) groups. The brittleness temperature is the highest low temperature when the pericarp is damaged by impact. The freezing medium (industrial ethanol) was injected into the cold well, and the intact seed was clamped vertically on the holder. The temperature was lowered in 1°C steps towards −40°C and the seed was held for 3.0 ± 0.5 min at each temperature point. The holder was lifted and the pericarp was impacted once within 0.5 s and each fruit observed to determine whether any damage had occurred. If the fruit was damaged, the temperature was increased back to room temperature. If the fruit was not damaged, the temperature was decreased and tested until the pericarp showed a brittle fracture upon impact.

### 2.14 Statistical analysis

SPASS 25.0 (SPASS Software Inc., SPSS, Chicago, IL, USA) was used to analyze the significance of the correlation between hulling time and percentage of damaged seeds, cellulose, hemicellulose, and lignin contents, total sugars and sugar components, X-ray diffraction spectrum, cellulose crystallinity, grain, and full width at half-maximum (FWHM), hardness, toughness, and brittleness temperature. Results were considered statistically significant at P ≤ 0.01. All figures were drawn with Origin Software (Originlab, Northhampton, MA, USA).

## 3 Results

### 3.1 Peeling efficiency and quality of manually and liquid N-treated seeds

#### 3.1.1 Peeling time and proportion of damaged seeds

**Figure 1:**
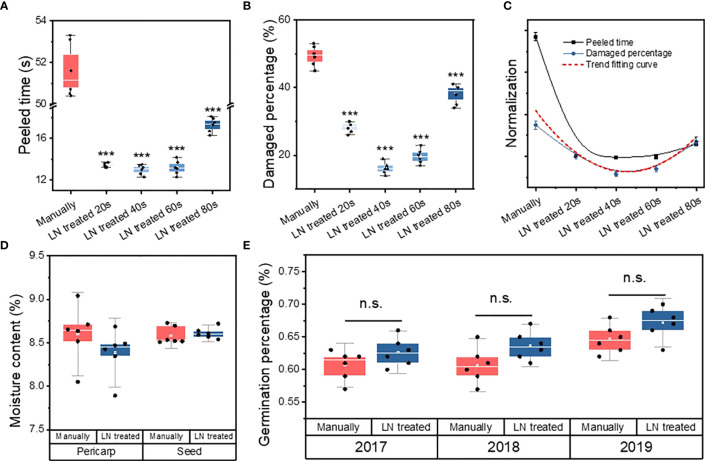
Peeled time **(A)**, percentage of damaged seeds **(B)**, and correlation fitting curve **(C)** of manually hulled seeds and those treated with liquid N for 20, 40, 60, and 80 s. Moisture content **(D)** and germination percentage **(E)** of manually hulled seeds and those treated with liquid N. Data represent the mean of six replicates ± standard deviation. “***” represents significant difference p<0.001, “ns” represents no significant difference.

The peeling time per seed was significantly longer in the manually hulled group (51.6 ± 1.31 s) than in the groups treated with liquid N for 20 s (13.4 ± 0.21 s), 40 s (13 ± 0.47 s), 60 s (13.2 ± 0.68 s), and 80 s (17.3 ± 0.69 s) (**Figure 1A**). The groups were ranked, from shortest hulling time to longest, as follows: manually hulled > 80 s liquid N > 20 s liquid N > 60 s liquid N > 40 s liquid N. Therefore, the fastest peeling time was in the group treated with liquid N for 40 s.

The percentage of damaged seeds in the manually hulled group (49% ± 3.0%) was significantly higher than that of seeds treated with liquid N for 20 s (28% ± 1.63%), 40 s (16% ± 1.7%), 60 s (20% ± 2.2%), and 80 s (38% ± 3.1%) (**Figure 1B**). The groups were ranked, from highest percentage of damaged seeds to lowest, as follows: manually hulled > 80 s liquid N > 20 s liquid N > 60 s liquid N > 40 s liquid N. The lowest percentage of damaged seeds was in the group treated with liquid N for 40 s.

A Pearson’s coefficient correlation analysis revealed a significant positive correlation between the peeling time and the percentage of damaged seeds (**Table 1**; **Figure 1C**). That is, the shorter the peeling time, the lower the level of damage. Because a shorter treatment resulted in less damage to seeds, a 40-s treatment with liquid N was selected as the appropriate method for subsequent experiments.

**Table 1:** Correlation analysis between peeled time and percentage of damaged seeds using SPSS.

		Hulled time (per fruit)	Damaged rate
**Hulled time (per fruit)**	Pearson Correlation	1	.835
	Statistical significance (two-sided)		.078
	Sums of Squares and Cross Products	1130.200	7.641
	Covariance	282.550	1.910
	N	5	5
**Damaged rate**	Pearson Correlation	.835	1
	Statistical significance (two-sided)	.078	
	Sums of Squares and Cross Products	7.641	.074
	Covariance	1.910	.019
	N	5	5

A positive Pearson’s coefficient indicates a positive correlation between the two parameters; a negative Pearson’s coefficient indicates a negative correlation between the two parameters.

With respect to other species with hard covering structures (such as *Sassafras tzumu, Cerasus yedoensis, Pinus bungeana, Armeniaca sibirica, and Pinus koraiensis*), we found that the covering structure can also be quickly removed after treatment with liquid N for an appropriate period of time, i.e. seconds (see **Table S1**, **Figure S1** available as **Supplementary Data**).

#### 3.1.2 Pericarp, seed moisture content, and seed germination percentage

Pericarp moisture content for the liquid N-treated group (8.4 ± 0.3%) was not significantly different from the manually hulled group (8.6 ± 0.3%) (**Figure 1D**). Similarly, the extracted seeds’ moisture content was the same after of liquid N-treatment (8.59 ± 0.1%) and manually hulling (8.61 ± 0.1%).

The germination of undamaged, full seeds in the manually hulled and liquid N-treated groups was 61% ± 2.25% and 63% ± 2.16%, respectively, in 2017; 61% ± 2.73% and 64% ± 2.16%, respectively, in 2018; and 65% ± 2.16% and 67% ± 2.48%, respectively, in 2019 (**Figure 1E**). The germination percentage was higher than 60% in both groups and was not significantly different between the two groups. Therefore, the liquid N treatment did not negatively affect the germination rate.

### 3.2 Cytoarchitecture and morphological characteristics of the pericarp in the manually hulled and liquid N-treated groups

#### 3.2.1 Cytoarchitecture of the pericarp

**Figure 2:**
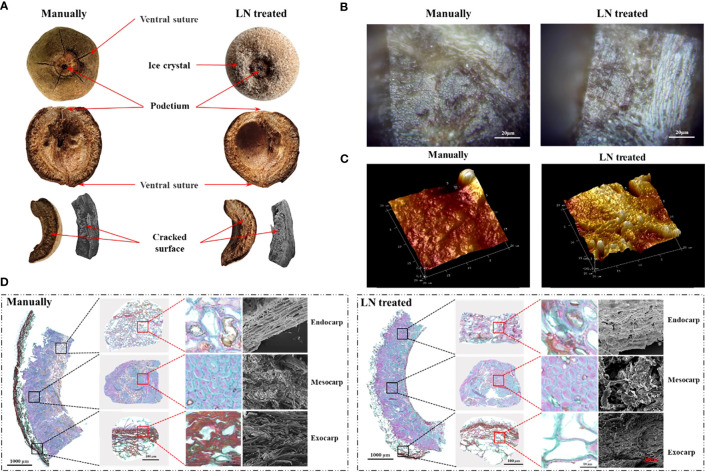
Anatomical structure **(A)**, mesocarp 2D morphology **(B)**, mesocarp 3D morphology **(C)**, and paraffin sections and scanning electron micrographs **(D)** of control and liquid nitrogen-treated fruits.

The pericarp is derived from the ovary wall (which forms from the carpel) that develops after fertilization. Each fruit had five ventral sutures, indicative of five carpels inside. The podetium was clearly visible. It cracked into five pieces along the abdominal sutures when knocked (**Figure 2A**).

Analyses of paraffin sections and by SEM showed the three layers of the pericarp; the exocarp, endocarp, and mesocarp. The exocarp was thin and composed of cork cells. The mesocarp had the largest volume and was composed of sclerenchyma cells (**Figure 2D**). The endocarp was composed of stone cells and a few parenchyma cells. In the exocarp of the manually hulled (control) group, the area of lignified cell walls (stained red) was significantly larger than that of cellulose cell walls (stained green). The surface was densely covered with slender cells with tips. However, in the group treated with liquid N, most of the densely slender cells had disappeared from the exocarp, and the area of lignified cell walls (stained red) was significantly decreased.

In the manually hulled group, the sclerenchyma cells of the mesocarp were closely arranged. The cell walls were composed of cellulose (stained blue-green) and lignin was present inside the cell. In the group treated with liquid N, the area of cellulose in cell walls in the mesocarp had not changed significantly, while that of lignin (stained red) was significantly increased. Large holes and gaps appeared between the cells.

In the manually hulled group, the endocarp cells were loosely arranged with holes of different sizes distributed on the surface. Cellulosic cell walls were dyed blue-green, and lignin in cell walls and inside the cells was dyed red. In the group treated with liquid N, the cells in the endocarp had become tightly arranged. The holes distributed on the endocarp surface had become larger and more numerous. The area of lignin in the cells was markedly increased (**Figures 2B–D**). Most of the exocarp was gone after the liquid N treatment. Large gaps and holes were present between the cells of the mesocarp and the endocarp. The area of lignin had decreased significantly in the exocarp but increased significantly in the mesocarp and endocarp.

#### 3.2.2 Morphological characteristics of the pericarp

At the crack along the ventral suture, the mesocarp was flat and regular in the manually hulled group, but it had an obvious undulating morphology in the liquid N-treated group. The structure of the cells was extremely irregular, with uneven distribution of raised and recessed parts. The highest raised part was 685.3 nm, and the lowest recessed part was −141.6 nm (**Figure 2C**). These results suggested that the pericarp cells contracted after being frozen with liquid N and did not return to their original shape on rewarming.

### 3.3 Contents of various substances in the pericarp

#### 3.3.1 Lignocellulose content in the pericarp

**Figure 3:**
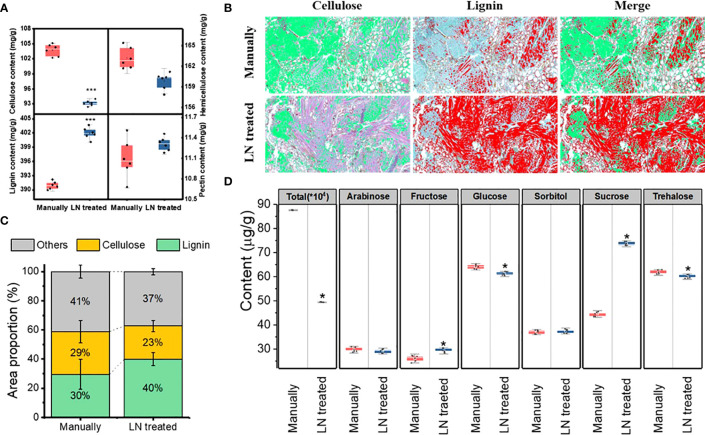
Lignocellulose content **(A)**, images **(B)** and proportion **(C)** of lignin and cellulose, sugar components **(D)** in the pericarp of the manually hulled and liquid nitrogen-treated groups. Data represent the mean of six replicates ± standard deviation. The cellulose is green and lignin red in **(B)**. Note: “*”represents a significant difference p<0.05, and “***” represents significant difference p<0.001.

The pericarp of *T. miqueliana* is mainly composed of plant cell walls, the basic components of which are cellulose, hemicellulose, lignin and pectin. The contents of cellulose, hemicellulose, lignin, and were significantly affected by the liquid N treatment. The contents of cellulose and hemicellulose decreased significantly (**Figure 3A**). The proportion of the area occupied by cellulose decreased from 29% to 23%. The lignin content increased significantly, and the proportion of the area occupied by lignin increased from 30% to 40%. The pectin content did not change significantly. Both cellulose and hemicellulose were partially degraded by the liquid N treatment. Compared with the manually hulled group, the group treated with liquid N showed increases in cellulose and hemicellulose degradation of 10.2% and 2.1%, respectively (**Figures 3B, C**). Thus, compared with hemicellulose, cellulose was more degraded by the liquid N treatment. A correlation analysis revealed that the degradation of cellulose and hemicellulose was negatively correlated with lignin content. The correlation coefficient between cellulose and lignin was –0.976, and that between hemicellulose and lignin was –0.763 (**Table 2**).

**Table 2:** Correlation analyses between cellulose, hemicellulose, and lignin by SPSS.

		Cellulose	Hemicellulose	Lignin
**Cellulose**	Pearson Correlation	1	.716**	-.976**
	Statistical significance (two-sided)		.009	.000
	N	12	12	12
**Hemicellulose**	Pearson Correlation	.716**	1	-.763**
	Statistical significance (two-sided)	.009		.004
	N	12	12	12
**Lignin**	Pearson Correlation	-.976**	-.763**	1
	Statistical significance (two-sided)	.000	.004	
	N	12	12	12

**, Significantly correlated at the 0.01 level (bilateral).

#### 3.3.2 Contents of total sugars in the pericarp

The liquid N treatment resulted in a significant change in the content of total sugars in the pericarp. For instant, glucose and trehalose decreased significantly, but arabinose and sorbitol contents did not change, but fructose and sucrose increased (**Figure 3D**). These results show that the liquid N treatment significantly affected the contents of total and individual sugars in the pericarp.

### 3.3 Mechanical properties of the pericarp

#### 3.3.1 Crystallinity, grain size, and full width at half-maximum of cellulose in the pericarp

When substances with a crystalline structure are X-rayed, diffraction occurs to form a characteristic XRD pattern. The cellulose in the pericarp has a crystalline structure, while other components do not. Therefore, it can be qualitatively analyzed by XRD.

**Figure 4:**
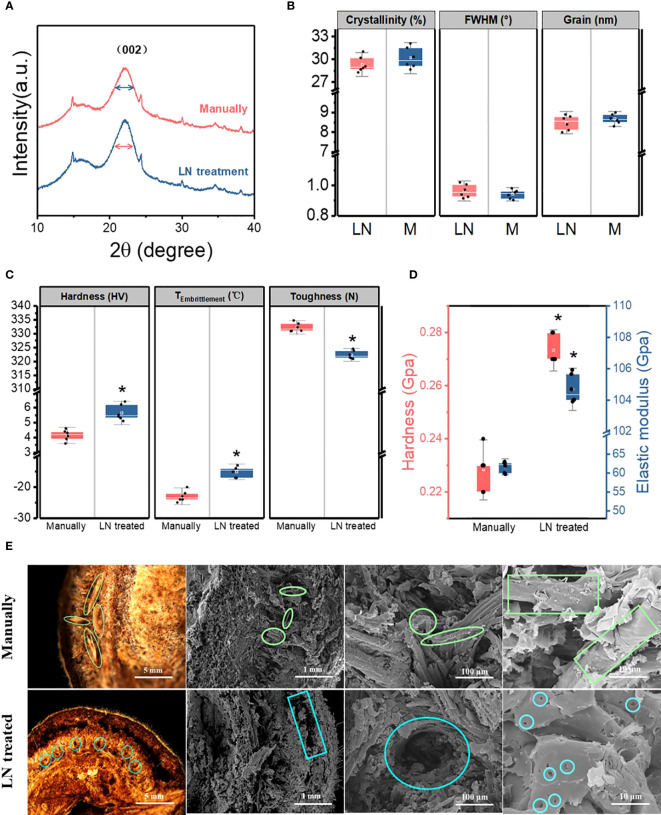
X-ray diffraction spectrum **(A)**, cellulose crystallinity, grain, and full width at half-maximum (FWHM) **(B)**, hardness, toughness, and brittleness temperature (°C) of pericarps from seeds hulled manually and those treated with liquid nitrogen **(C)**. Hardness and elastic modulus **(D)** and morphological characteristics of the pericarp at the cracked surface of the ventral suture of manually hulled and liquid N-treated groups **(E)**. Data represent the mean of six replicates ± standard deviation.

The cellulose in the pericarp had similar X-ray diffraction patterns in the two groups (manually hulled and liquid N-treated groups). The 2θ diffraction angle at 22.1° was the characteristic peak of cellulose crystal planes in both groups, indicating that the liquid N treatment did not change cellulose structure. The distance between crystal layers did not change, providing further evidence that cellulose structure was unchanged. The full width at half-maximum (FWHM) of the cellulose (002) crystal plane characteristic peak was 0.942 ± 0.03° and 0.963 ± 0.04° in the manually hulled group and the liquid N-treated group, respectively. Thus, the liquid N treatment slightly increased the FWHM. The crystallinity of cellulose refers to the percentage of the crystalline area composed of cellulose out of total cellulose, which reflects the integrity of crystallization (Wu and McGinity, 1999). The crystallinity of cellulose was not significantly higher in the manually hulled group (30.2% ± 1.4%) than in the liquid N-treated group (29.3% ± 1.1%). The grain size of cellulose (002) was also unchanged on treatment (8.67 ± 0.26 nm and 8.48 ± 0.38 nm in the manually hulled and liquid N-treated groups respectively) (**Figures 4A, B**). Thus, the liquid N treatment slightly decreased the grain size and crystallinity of cellulose in the pericarp.

#### 3.3.2 Hardness, toughness, and brittleness of the pericarp

The average hardness of the pericarp in the manually hulled group and the liquid N-treated group was 4.15 ± 0.36 and 5.65 ± 0.52 HV, respectively; the average toughness was 332.25 ± 1.61 and 322.36 ± 1.51 N, respectively; and the average brittleness temperature was −23 ± 1.78°C and −15 ± 1.67°C, respectively (**Figure 4C**). These results show that the liquid N treatment significantly increased the hardness and brittleness temperature of the pericarp, and significantly decreased the toughness. Thus, the liquid N treatment changed the mechanical properties of the pericarp.

#### 3.3.3 Hardness and elastic modulus of the mesocarp at the cracked surface of the ventral suture

The nanoindentation test indicated that the hardness of the mesocarp at the cracked surface of the ventral suture in the manually hulled group and liquid N-treated group was 0.22 ± 0.01 and 0.27 ± 0.01 GPa, respectively; and the elastic modulus was 50.91 ± 1.45 and 107.5 ± 0.89 GPa, respectively (**Figure 4D**). These results show that the liquid N treatment significantly increased both the average hardness and elastic modulus of the mesocarp at the cracked surface of the ventral suture.

#### 3.3.4 Morphology of the pericarp at the cracked surface of the ventral suture

The pericarp cracked along the same ventral suture in both groups. In the manually hulled group, severed vascular bundles were distributed on the cracked surface. The spiral vessel of the vascular bundle emerged after it separated from cells under the impact load. The exocarp and mesocarp were separated in seeds treated with liquid N and there was an obvious gap between them. Many small holes appeared on the surface of the mesocarp. After liquid N treatment, the binding force between the cells became weak, so that the cells separated easily under an impact load (**Figure 4E**).

## 4 Discussion

We hypothesized that liquid N treatment of *T. miqueliana* pericarps would result in sufficient stress and strain to compromise cell macro- and micro-structure, thereby making seed extraction easier without potentially compromising seed viability.

### 4.1 Pericarp cell structure

In this study, in both the manually hulled and liquid N-treated groups, the pericarp split all along the ventral suture. However, the morphology of the split surface differed between the two groups. In the manually hulled group, the spiral vessel of the vascular bundle was pulled out after it separated from cells under an impact load. In the liquid N-treated group, the exocarp and mesocarp were separated, and the surface of the mesocarp was covered with small holes. Similarly, perilla seeds subjected to freeze-thaw (-20°C) had damaged seed coats, with signs of splitting at the seams, that facilitated seed processing and the efficient extraction of perilla seed oil (Lee et al., 2020). In another industrial application, liquid N freeze-drying can help to fabricate cellulose nanofiber (CNF). Treatment results in the destruction of the 3-Dcellulose network such that the CNF produced had a porous layered structure (Wang et al., 2020).

We used AFM to show that the mesocarp crack along the ventral suture was smooth in the manually hulled group, but undulating and rough in the liquid N treated group with an uneven distribution of raised and recessed parts. These findings suggest that the pericarp cells that contracted during liquid N treatment did not fully expand after rewarming. When wheat seeds were given a prolonged cold plasma treatment in an attempt to improve seed wettability, the seeds had enhanced seed coat roughness (as shown by AFM) but the germination of seedlings was slowed (Starič et al., 2022). In contrast, liquid N-treated *T. miqueliana* fruits had rougher morphology without comprised seed viability.

### 4.2 Mechanical properties of the pericarp

In the present study, the size of the intensities of the characteristic peaks, cellulose grains and overall crystallinity were reduced in the pericarp after LN treatment. Crystallinity is an important factor in determining the cellulose hydrolysis efficiency (Alvira et al., 2010). Shen et al. (2019) found that the intensities of the characteristic peaks decreased after liquid N and ball milling (LN–BM) pretreatments. The crystallinity index (CI) of cellulose significantly decreased to 41.7% after only 2 h of LN−BM treatment and further decreased to 39.4% after 3 h of LN−BM treatment. LN−BM was effective at destroying the hydrogen bond networks within cellulose (Shen et al., 2019). It is possible that liquid N-treatment also reduced the hydrogen bond networks within cellulose in the pericarp of *T. miqueliana*.

Low temperature brittleness refers to the impact absorption energy of the material as the temperature decreases. Below a certain temperature, the impact absorption energy decreases significantly and the material changes from a ductile state to a brittle state (Diao et al., 2017). After the liquid N treatment, the hardness and modulus of elasticity at the ventral suture, and the hardness and brittleness temperature of the pericarp were significantly increased, while the toughness was significantly decreased. This response is similar to the low temperature processing of walnut fruits, such that the maximum effect on the destruction of the hazelnut shell is achieved by freezing it in nitrogen for 15 minutes, the load being about 208.3 N, which differ much from the load without freezing and is F = 261 (N). Similarly, hazelnut shells become fragile and easily destroyed when the nuts are exposed to shock deformations, whilst the integrity of the nut kernels is preserved as much as possible (Neverov et al., 2022). Thus it seems that liquid N treatment dramatically alters *T. miqueliana* pericarp mechanical properties, including changing the fracture type from ductile to brittle.

### 4.3 Pericarp chemical composition

Pericarps are rich in plant secondary cell walls polymers, such as cellulose, hemicelluloses, and lignin (Landucci et al., 2020). These ubiquitous organic polymers are non-toxic, biodegradable (in time), provide mechanical properties and have slightly different water holding properties, such that hemicellulose > cellulose > lignin (Santos et al., 2013).

The main building blocks of lignin are the hydroxy-cinnamyl alcohols (or monolignols) coniferyl alcohol and sinapyl alcohol (Vanholme et al., 2010). Cellulose is composed of glucose units (Hu et al., 2015). Hemicelluloses are heterogeneous polymers of pentoses (xylose, arabinose), hexoses (mannose, glucose, galactose), and sugar acids (Gunnarsson et al., 2008). In this study, the composition of the pericarp on a per weight basis in the control (manually dehulled) group was 10.4% cellulose, 16.3% hemicellulose, 39.1% lignin, and 1.1% pectin. Although these compounds are deemed to be relatively stable, after liquid N treatment, the pericarp contents of cellulose and hemicellulose decreased significantly, whilst that of lignin increased significantly, and that of pectin did not change. It is known that the polymorphic structures of cotton cellulose can change instantaneously when ammonia and alkali-impregnated fabrics are immersed in liquid N, e.g., by conversion to cellulose III or to cellulose II (Jung et al., 1977). Similarly, when cellulose is pretreated with liquid N coupled with ball milling (LN–BM), the glucose yield from cellulose increases almost two-times compared to that obtained from untreated cellulose (Shen et al., 2019). We assume, therefore, that the observed low temperature-induced modifications of cellulose and glucose contents in *T. miqueliana* pericarp may reflect similar degradation processes.

### 4.4 Post-liquid N treatment seed quality attributes

Seed banking under dry (c. 3–7% moisture content) and cold (i.e., −18°C or in liquid N) conditions underpins ex situ plant conservation; it is cost effective and requires less space compared to in-situ methods (Li and Pritchard, 2009). To survive transfer to sub-zero temperature, seeds must be dried below the high moisture freezing limit (HMFL), which is related to seed oil content (SOC), being about 9% moisture content (MC) for a 50% SOC and just over 20% MC for very low SOC (Pritchard, 1995). In this study, the extracted seeds’ MC was the same after of liquid N-treatment (8.59 ± 0.1%) and manually hulling (8.61 ± 0.1%). Although the SOC was not determined in *T. miqueliana*, *Tilia* species tend to produce desiccation tolerant seeds (Seed Information Database; http://data.kew.org/sid/) with low SOC, e.g. 18% average for three species (Plant FA database; https://plantfadb.org/). It is likely, therefore, that *T. miqueliana* seeds at 8.5% MC were below the HMFL when exposed to liquid N and that there was insufficient water in the seeds for damaging ice crystals to form. This is supported by observation that the seeds germinated normally after liquid N treatment. This response is consistent with the successful cryopreservation of *Tilia cordata* seeds at 7 – 18% MC (Chmielarz, 2002). Such cryotolerance in seeds has been observed in many species. For example, seed viability of 89% of 103 plant species from 33 families from five regions of the Russian Far East did not decrease in the course of cryogenic storage (Kholina and Voronkova, 2008). Similarly, it was concluded that a large proportion of native Western Australian species may be amenable to storage in liquid nitrogen based on the screening of 90 species from 33 families (Touchell and Dixon, 1993). Thus, transfer of seeds to liquid N can be an effective means of plant conservation.

In addition to inducing changes in the microstructure of the pericarp, liquid N treatment of seeds might cause the formation of cracks in the seed coat or covering structures that later facilitate the ingress of water and improved germination (Busse, 1930; Brant et al., 1971; Normah and Vengadasalam, 1992; Salomao, 1995). In tropical orthodox seed, liquid N treatment had no adverse effect on the germinability of seeds with heterogeneous levels of hardness (Salomão, 2002). In contrast, liquid N treatment had partially beneficial effects on *B. basiloba*, *S. brasiliensis*, *S. mombin, Bowdichia virgilioides*, *Pterodon emarginatus* (*Fabaceae*) and *Apeiba tibourbou* (Tiliaceae) seeds (Salomão, 2002). In corroboration, the liquid N treatment dehulling method did not have a negative effect on *T. miqueliana* seed germination, with germination being > 60% in every tested year. The widescale use of liquid N treatment/storage for biodiverse species suggested to us that the liquid N dehulling treatment might be a safe procedure to test on other forestry species with propagules that are difficult to process.

### 4.5 Potential for liquid N treatment of hulling

A nut is a fruit composed of a hard shell and a seed, where the hard-shelled fruit does not open to release the seed. For most of the nut fruits the common post-harvest processes are drying, shelling and storage (Ashok Raj, 1979). The most common traditional method for shelling the nut fruit is shelling by hand (manual), which is a time-consuming process and labour-intensive method. Based on our understanding of the impacts of liquid N treatment on pericarp structural properties, we hypothesized that a liquid N treatment could be used to facilitate the extraction of *T. miqueliana*, seeds, and those of other species. However, it was important to establish that the seeds would not be negatively affected by this treatment.

We selected three species that have seeds with mechanical dormancy: *Sassafras tzumu* (Lauraceae) (Zhou et al., 2006), *Pinus bungeana* (Pinaceae), which is suitable for landscaping because of its unique white bark (Guo et al., 2019), and *Cerasus yedoensis* (Rosaceae), which has brightly coloured flowers and is of high ornamental value (Wu, 1985). We also choose a wild apricot (*Armeniaca sibirica*, *Prunus* sp.), which is an important local fruit and potential industrial crop (Rai et al., 2016; Hansakon et al., 2019; Qin et al., 2019), and *Pinus koraiensis* (Pinaceae), which is one of the most economically important tree species in Northeastern China, with pine nut exports generating > US $ 250 million dollars each year (Lin et al., 2018) and other attributes also being of importance, such as the pine nut shell as a potential source of bioenergy (Kaviriri et al., 2021). The results for all six species studied show that the peeling time and percentage of damaged seeds is lower (<20% of seeds) after liquid N treatment than after manual hulling. Overall, this method has similar efficiency to the machine shelling of cashew (Ojolo et al., 2010). Nonetheless, liquid N shelling is more economical and greatly reduces the cost of hulling (see **Table S2** available as **Supplementary Data**).

## 5 Conclusion

This is the first report of the use of liquid N to help remove the hard pericarps of *T. miqueliana*. On the basis of hulling time and seed damage percentage, a 40-s treatment with liquid N was identified as the best method. This method was fast and caused the least damage to the seeds, and it did not negatively affect germination. Analyses of the microstructural and mechanical properties of the pericarps revealed, for the first time, the mechanism of the liquid N treatment. Analyses of paraffin sections and the SEM and AFM results showed that the liquid N treatment destroyed the cellular structure of the pericarps. There were significant changes in lignocellulose content, with a significant increase in lignin content and a significant decrease in cellulose and hemicellulose contents in the pericarps in the liquid N-treated group. The nanocompression constant, Vickers hardness, and brittleness results revealed changes in the mechanical properties of the pericarp from ductile to brittle, with a significant increase in hardness and brittleness and a significant decrease in toughness. Thus, this study shows that the liquid N hulling method could support large-scale, economical production of seeds and seedlings for breeding, forestry and conservation/restoration. Furthermore, it also provides a new idea for postharvest treatment and pre-treatment of deep processing in nuts.

## Data availability statement

The original contributions presented in the study are included in the article/**Supplementary Material**. Further inquiries can be directed to the corresponding author.

## Author contributions

YS conceived the original screening and research plans; YW and CP designed and performed all experiments; YW and XS analyzed the data; YW conceived the project and wrote the article with contributions of all the authors. AV, ZD and HP revised the article. MW performed supplementary experiments. YW agrees to serve as the author responsible for contact and ensures communication. All authors contributed to the article and approved the submitted version.
