# Supplementary material for: Cryo-attenuated properties of Tilia miqueliana pericarps and seeds
Source: Front Plant Sci. 2023 Aug 15;14:1228069. doi: 10.3389/fpls.2023.1228069 (PMC10486270; doi:10.3389/fpls.2023.1228069)
Supplement: Supplementary file 1 [file DataSheet_1.docx]

**Table 1** The functional model coefficients, goodness of fit R2, F-statistic values and p-values of the hulled time and percentage of damaged seeds for *Sassafras tzumu* (A), *Cerasus yedoensis* (B), *Pinus bungeana* (C), *Armeniaca sibirica* (D), and *Pinus koraiensis* (E) seeds.

| **Numer** | **Formula** | **Fitting parameters** | **R^2^ statistic** | **F statistic** | **p value** |
| --- | --- | --- | --- | --- | --- |
| **Fig 1**  **Red curve** | y = p1+p2*x+p3*x^2+p4*x^3+p5*x^4+p6*x^5 | p1 = 4.408674435  p2 = -0.176927471  p3 = 0.019078932  p4 = -0.000879065345  p5 = 1.84228407e-05  p6 = -1.40204774e-07 | 0.999726104 | 32120.141632557 | 3.56995165e-77 |
| **Fig 1**  **Blue curve** | y = p1+p2*x+p3*x^2+p4*x^3+p5*x^4+p6*x^5 | p1 = -0.155771705  p2 = 0.100782551  p3 = -0.010605694  p4 = 0.000497541806  p5 = -1.05382585e-05  p6 = 8.2931506e-08 | 0.996859730 | 2793.506605018 | 7.19863654e-54 |
| **Fig 2**  **Red curve** | y = p1+p2*x+p3*x^2+p4*x^3+p5*x^4+p6*x^5 | p1 = 3.936856848  p2 = 0.00603623965  p3 = 0.000378929  p4 = -3.35635437e-06  p5 = 1.32452464e-07  p6 = -1.05961971e-09 | 0.999997664 | 3766491.334790151 | 1.08122749e-122 |
| **Fig 2**  **Blue curve** | y = p1+p2*x+p3*x^2+p4*x^3+p5*x^4+p6*x^5 | p1 = 0.058839827  p2 = 0.00294121845  p3 = -0.000178488  p4 = 9.71254004e-06  p5 = -2.02170961e-07  p6 = 1.57961251e-09 | 0.999995697 | 2045216.605435306 | 7.38321716e-117 |
| **Fig 3**  **Red curve** | y = p1+p2*x+p3*x^2+p4*x^3+p5*x^4+p6*x^5 | p1 = 3.568419348  p2 = -0.0700118754  p3 = 0.007847771  p4 = -0.000335110023  p5 = 7.19427739e-06  p6 = -5.52711619e-08 | 0.999992911 | 1241307.732109181 | 4.35646242e-112 |
| **Fig 3**  **Blue curve** | y = p1+p2*x+p3*x^2+p4*x^3+p5*x^4+p6*x^5 | p1 = -0.165092114  p2 = 0.0843819405  p3 = -0.008867922  p4 = 0.000413786818  p5 = -8.6938681e-06  p6 = 6.79051956e-08 | 0.997809281 | 4008.146167981 | 2.61502288e-57 |
| **Fig 4**  **Red curve** | y = p1+p2*x+p3*x^2+p4*x^3+p5*x^4+p6*x^5 | p1 = 3.513683890  p2 = 0.0682201744  p3 = -0.002109537  p4 = 0.000101793359  p5 = -2.22107646e-06  p6 = 1.77832629e-08 | 0.999999636 | 24174216.721391898 | 1.86550967e-140 |
| **Fig 4**  **Blue curve** | y = p1+p2*x+p3*x^2+p4*x^3+p5*x^4+p6*x^5 | p1 = 0.104581529  p2 = 0.0253886009  p3 = -0.003493388  p4 = 0.000180266975  p5 = -3.97502996e-06  p6 = 3.20381874e-08 | 0.999240523 | 11578.122363551 | 1.98053584e-67 |
| **Fig 5**  **Red curve** | y = p1+p2*x+p3*x^2+p4*x^3+p5*x^4+p6*x^5 | p1 = 2.637927510  p2 = 0.0681190396  p3 = 0.002772995  p4 = -0.000185097014  p5 = 4.20182255e-06  p6 = -3.42834059e-08 | 0.999999751 | 35276590.273730576 | 4.5692666e-144 |
| **Fig 5**  **Blue curve** | y = p1+p2*x+p3*x^2+p4*x^3+p5*x^4+p6*x^5 | p1 = 0.157038060  p2 = -0.0215968383  p3 = 0.002531055  p4 = -0.000120792225  p5 = 2.58039254e-06  p6 = -1.99809164e-08 | 0.999849555 | 58484.199315272 | 6.73247738e-83 |

R^2^ close to 1 indicates a good model fit.

**
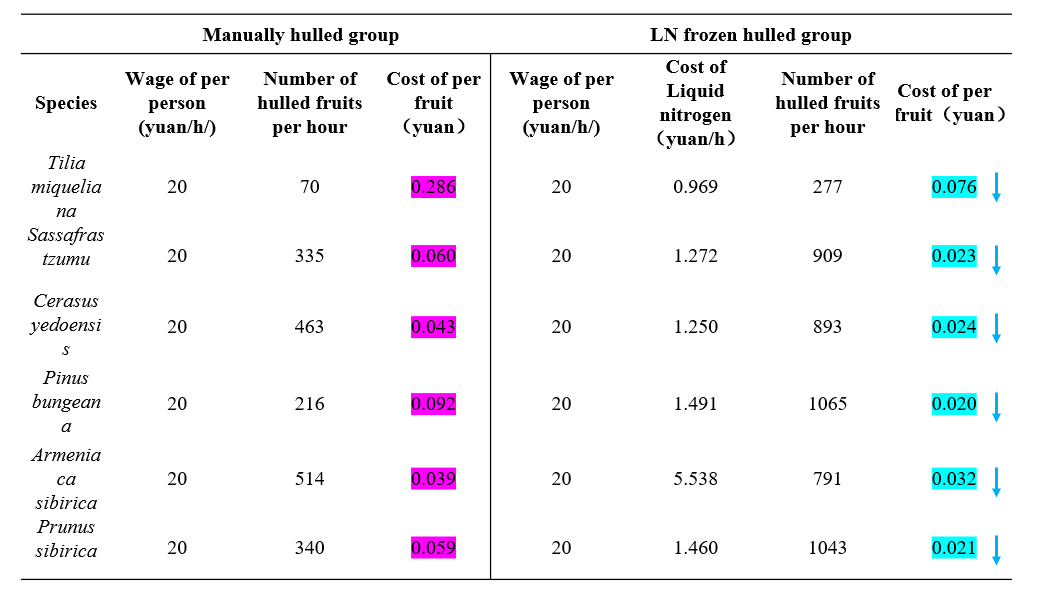
Table 2** Wage of per person, number of hulled fruits per hour, cost of per fruit of manually hulled group, and LN frozen hulled group for *Tilia miqueliana*, *Sassafras tzumu*, *Cerasus yedoensis*, *Pinus bungeana*, *Armeniaca sibirica*, and *Pinus koraiensis*.


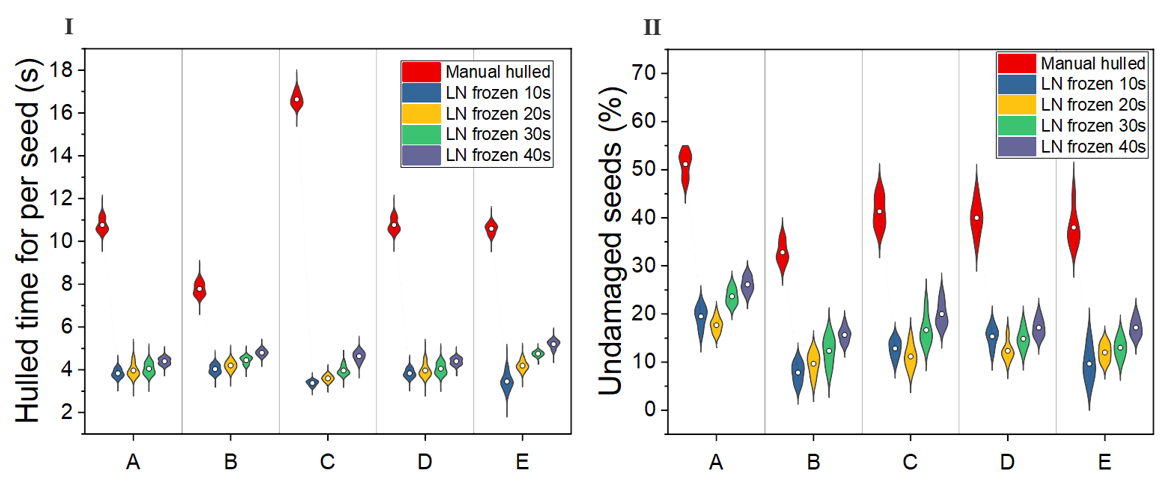
**Fig. 1.** Hulled time (I), percentage of damaged seeds (II) for *Sassafras tzumu* (A), *Cerasus yedoensis* (B), *Pinus bungeana* (C), *Armeniaca sibirica* (D), and *Pinus koraiensis* (E).
